# An Insight into Chip and Surface Texture Shaping Under Finish Turning of Powder Steels Infiltrated with Tin Bronze

**DOI:** 10.3390/ma17246244

**Published:** 2024-12-20

**Authors:** Kamil Leksycki, Eugene Feldshtein, Larisa Dyachkova, Katarzyna Arkusz, Maciej Ceglewski, Łukasz Czerwiec

**Affiliations:** 1 Institute of Mechanical Engineering, University of Zielona Gora, 4 Prof. Z. Szafrana Street, 65-516 Zielona Gora, Poland; e.feldsztein@iim.uz.zgora.pl (E.F.); k.arkusz@iim.uz.zgora.pl (K.A.); maciekceglewski@gmail.com (M.C.); czerwiec.ukasz@gmail.com (Ł.C.); 2 The State Scientific Institution “Power Metallurgy Institute”, Belarusian National Academy of Sciences, 41 Platonov Street, 220005 Minsk, Belarus; dyachkova@tut.by

**Keywords:** power steel, infiltration, finish turning, chip shaping, chip spreading ratio, surface texture

## Abstract

The manufacturing of work parts made of powder (sintered) steels is currently widespread in industry, as it provides minimal processing allowances and high dimensional accuracy, as well as the required properties and unconventional chemical composition. At the same time, their low tensile or bending strength must be considered a serious disadvantage. In order to minimize these disadvantages, a number of strengthening technologies are used, among which is the infiltration of porous base materials with metal alloys. In this study, the details of finish turning of sintered iron-graphite-based steel infiltrated with tin bronze with molybdenum disulfide addition are considered. Changes in the shape of chips and their geometric features, as well as the 3D parameters and topography features of the surface machined, are presented after finish turning with AH8015 carbide inserts. The cutting speed (*v_c_*) and feed rate (*f*) were used as variable parameters. It was found that when turning the powder steels under study, the chips took the shape of small fragments or element chips, including segmented chips. For quenching steel, the formation of irregular lamellae was observed and for the initial state, a serrated chip was registered. For the initial state, a reduction in *K_b_* values was observed in the range of the *v_c_* of 50–100 m/min and *f* of 0.05–0.075 mm/rev, and for quenching in the range of 225–250 m/min and 0.05–0.075 mm/rev. Compared to the initial state, for quenching, depending on the cutting parameters, a 14% reduction in the chip spreading ratio *K_b_* or an increase from 2 to 32% was registered. For the initial state and quenching, a decrease in the *Sp* and *Sv* parameters was achieved in the range of the *v_c_* of 200–250 m/min and *f* of 0.05–0.075 mm/rev, and there was an increase in the range of 50–150 m/min and 0.125–0.15 mm/rev. Compared to the initial state, an increase in the *Sz* parameter from 10 to 35% was observed for quenching. On the surfaces machined with *v_c_* = 50 m/min and *f* = 0.05 mm/rev, waves and single significant peaks were observed. On the other hand, *v_c_* = 250 m/min and *f* = 0.15 mm/rev provided classical feed tracks in the form of valleys and irregular ridges on the surfaces machined. The test results can be useful in the design and manufacturing of industrial parts made of powder steels.

## 1. Introduction

Components manufactured with sintered powder materials are now widely used in industry. Such products are characterized by major advantages, including shapes that are very close to the final shapes (minimal machining allowances), high dimensional accuracy, certain properties (high hardness and wear resistance, good fatigue strength), and an unconventional chemical composition. At the same time, the low tensile or bending strength of the sintered materials has to be considered as a significant deficiency. In order to minimize these disadvantages, a number of strengthening technologies are being used; among them is the infiltration of the base porous materials with metal alloys or quenching.

There are multiple research studies known in the mentioned areas. For example, Damin et al. [1] studied the addition of molybdenum as an alloying element in a self-lubricating Fe + 0.6%C + 3%SiC composite. Molybdenum enrichment provided a significant increase in the abrasion resistance and a decrease in the average coefficient of friction. Markovsky et al. [2] reinforced a Ti-6Al-4V alloy with TiC particles. A post-sintering solution treatment and water quenching followed by aging were used. The matrix and reinforcing phase underwent pronounced structural changes during the thermal treatment, which improved the material’s mechanical properties. Satish et al. [3] analyzed the effect of Al_2_O_3_ and MoS_2_ inclusions in a plasma-sprayed aluminum coating on the microstructure, mechanical, and wear properties. It was found that increasing the amount of Al_2_O_3_ and MoS_2_ in the coating reduced the wear rate and coefficient of friction (CoF) significantly, and this could be attributed to the increased hardness and lubricity characteristics of the reinforcing elements. Wang et al. [4] investigated the tribological behaviors of lubricating oil with Al_2_O_3_ and MoS_2_ particles for Mg alloy. Al_2_O_3_ particles resulted in a more stable anti-wear surface, while MoS_2_ additives formed a film with MoO_3_ and MgSO_4_ compounds. Selvan et al. [5] fabricated AA2219/B_4_C/Gr/MoS_2_ hybrid composites using MoS_2_ and graphene for secondary reinforcement and defined that the hardness and wear resistance of the composites depended on the percentage of Gr and MoS_2_, the mixing rate, and the pouring temperature, providing excellent final composite properties. Tabrez et al. [6] studied nickel composites reinforced with different solid lubricants and showed their effects on the microstructure, yield strength, tensile strength, plasticity, hardness, friction, wear, and fatigue. Rumi and Rahman [7] investigated the influence of heat treatment modes on the microstructure, microhardness, and electrical conductivity of an aluminum composite reinforced with Al_2_O_3_ nanoparticles. The materials were treated using quenching and subsequent artificial aging. The heat treatment increased the hardness and electrical conductivity of the composite. Stepanova et al. [8] studied the influence of Al, Cu, and Mn on the structure, mechanical, and tribotechnical properties of cast irons. The alloying resulted in the formation of a structure in which pearlite was accompanied by micro-volumes of martensite and retained austenite. Cu particles were present both within colonies of lamellar pearlite and within martensite crystals. The presence of martensite in the structure of cast iron increased its wear resistance. So, metal matrix composites (MMCs) reinforcement provided a definite improvement in the microstructural, mechanical, and operational properties.

MMCs are related to a group of materials that are difficult to cut due to their high content of fine hard inclusions, heterogeneous structure, and special mechanical and thermal properties. Some results from studies regarding their machinability are described below. Karabulut [9] investigated the impact of milling parameters for AA7039/Al_2_O_3_ composites on the cutting forces and surface roughness. He revealed that the material structure had the most significant effect on the surface roughness, and the feed rate had the most significant effect on the cutting forces, while the cutting speed had the least significant effect. Shoba et al. [10] examined the effect of the depth of cut, feed rate, and cutting speed on the components of cutting forces when machining MMCs with SiC and rice husk ash additives. All the cutting forces components were decreased with increasing reinforcement percentage. When the composites with a low percentage of reinforcement were machined at large depth-of-cut and high speeds, a built-up edge (BUE) was formed. The cutting forces also decreased as the cutting speed decreased and the feed rate and depth-of-cut increased. Kumar et al. [11] analyzed the effect of the cutting speed and feed rate on the cutting forces and surface roughness during the dry turning of nanohybrid composites based on an Al2219 alloy with n-B_4_C and n-B_4_C/MoS_2_ particles. It was revealed that increasing the feed rate and adding n-B_4_C particles increased the cutting forces and surface roughness for the nanocomposites, and that they decreased with an increasing cutting speed. When the n-B_4_C/MoS_2_ composite was machined with a lower cutting speed, a BUE was observed. Tomadi et al. [12] described the effect of milling parameters on the surface roughness of AlSi/AlN MMC. Experiments were carried out within a wide range of cutting parameters under dry machining conditions. The optimum machining conditions were found. Teng et al. [13] performed modeling using the finite element method to analyze interactions between the tool and composite particles, the process of chip formation, and changes in the cutting forces and stresses distribution in the workpiece depending on the uncut chip thickness. The modeling revealed the appearance of segmented chips. Duan et al. [14] introduced a model of chip formation and changes in cutting forces for different stages of Al/SiC_p_ MMCs machining. The chip morphology was classified as segmented. Gopal et al. [15] studied the effect of material and milling parameters on the force components, cutting temperature, and surface roughness under face milling of Mg MMC with cemented carbide tools. Jiang et al. [16] examined the machinability of TiB_2_/Al MMCs and showed that the cutting forces for MMCs were greater than that of an unreinforced alloy and mainly depended on the feed rate; the residual stresses in MMCs were compressive, while they were almost equal to zero in unreinforced alloys; and the surface roughness for MMCs was less than that of unreinforced alloys at the same cutting speed. However, the opposite result was observed when the feed rate was increased. Xiong et al. [17] studied the chip formation process for orthogonal cutting of TiB_2_/7050Al MMC and showed that segmented chips usually appeared at different speeds and feed rates, but they were different compared to when cutting unreinforced aluminum alloy. Niknam et al. [18] described the turning of titanium metal matrix composites (Ti-MMC) under dry and semi-dry modes. Higher values of cutting forces were observed with semi-dry modes, because the formation of a thin lubricant film prevented the smooth advancement of the cutting tool in the workpiece. Higher *Ra* and *Rq* values were observed at a higher cutting speed. Liao et al. [19] presented a detailed review of MMCs machining with a main focus on the aspects related to the surface integrity of the workpiece, the influence of the mechanical and microstructural properties of the material, and the material removal mechanism on the surface quality as well as the fatigue properties of the part machined. Salur et al. [20] performed experiments for MMCs’ dry drilling with different feeds. Composites based on CuSn10 bronze with a GGG-40 spheroid graphite cast iron additive were manufactured under different temperatures, pressures, and reinforcement ratios, and their effect on the axial force and roughness of the surface machined under different feeds was evaluated. Ajithkumar and Xavior [21] presented the effect of cutting parameters on the surface roughness and cutting forces when dry machining Al7075-10%SiC-0.1% B_4_C, Al7075-10%SiC-0.1% graphene, and Al7075-10%SiC-0.1% CNT composites using different tools. The feed rate was a key factor influencing the surface roughness. The presence of graphene showed the minimum surface roughness compared to other additives. The depth-of-cut was the most influential factor on the cutting forces for all composites. Kim et al. [22] analyzed ultrasonically assisted turning and conventional turning of SiC_p_/Al MMC under minimum quantity lubrication (MQL). The ultrasonically assisted turning improved both the cutting forces and surface topography. Deǧirmenci et al. [23] examined the effect of cooling/lubrication and cutting parameters during the milling of hybrid Al-4Gr composites obtained by adding WC and Al_2_O_3_ in different percentages. The surface roughness, chip morphology, and cutting temperature were evaluated for different cooling/lubrication conditions. Chakravarthy et al. [24] described the characteristics of cryogenic coolant and its effect on the axial force, burr height, cutting temperature, and surface roughness under drilling of Al-matrix composites reinforced with nano SiC particles compared to dry machining. A reduction of the friction in cryogenic LN_2_ resulted in a lower surface roughness and temperature. Ravikiran et al. [25] considered three types of CNT-reinforced aluminum metal matrix composites (Al MMCs). The temperature, surface roughness, material removal rate, power, thrust force, torque, and diametric error were analyzed. The chip morphology under different drilling conditions was also studied. Kulkarni et al. [26] analyzed the turning of unsintered and sintered compacts prepared from FLC-4608 powder steel. When machining unsintered materials, reducing the feed rate impacted the conditions of powder particles’ peeling during the material removal and improved the quality of the surface machined. Increasing the cutting speed and feed rate was found to reduce the size of exit-edge breakout. The turning of unsintered materials was characterized by lower cutting forces and a favorable chip morphology. Niu et al. [27] studied the peculiarities of the orthogonal turning of compacted graphite iron (CGI) on the chip formation, its morphology, and the microstructures of the chip roots. It was found that the specific CGI behavior affected the microstructure evolution in the cutting deformation zone, including significant changes in the plate structure of pearlite, grain refinement, and the accumulation of the chip dislocation density. Sivalingam et al. [28] fabricated hybrid Al MMCs with reinforcing particles of TiO_2_ and Gr, and studied their machinability under dry, MQL with vegetable oil, cryogenic CO_2_, and hybrid (MQL + CO_2_) machining conditions. Higher cutting forces and surface quality degradation were observed when dry milling Al MMCs. The combination of MQL + CO_2_ cutting media was more effective, reducing cutting forces by 39–28% compared to the other media. The surface roughness was reduced with favorable chip formation. Huan et al. [29] presented the modeling of particle-reinforced Ti MMCs. Simultaneously, turning tests were carried out to verify the model accuracy in terms of predicting cutting forces and material removal processes based on the cutting forces, chip microstructure, and particle fracture patterns. The cutting speed and cutting forces were directly proportional. The depth-of-cut had a significant effect on the cutting forces.

It can be summarized that the cutting process of MMCs based on various metals and their alloys reinforced with fine hard or anti-friction additives differs considerably compared to the cutting of base materials. In the references presented above, serious changes were registered in the area of chip shaping, in the values of forces and temperatures, and in the surface integrity parameters. The aim of this survey was the analysis of chip shaping, the chip spreading ratio, and changes in the surface topography of Fe-Gr powder steels infiltrated with tin bronze, reinforced with molybdenum disulfide and machined using dry finish turning with variable cutting speeds and feed rates.

## 2. Materials and Methods

### 2.1. Materials

The analyzed materials were made of powder steel skeleton under infiltration by CuSn5 tin bronze with some additives, including MoS_2_ particles. Fe-base green skeleton contained 1.2% graphite addition and complete infiltration technology was described by Dyachkova and Feldshtein [30]. The final material can be designated as FeGr1.2Cu17Sn1(MoS_2_)1. It was tested in two grades: initial state and quenching state. Details of the sample fabrication and their dimensions and properties were described by Leksycki et al. [31].

**Figure 1:**
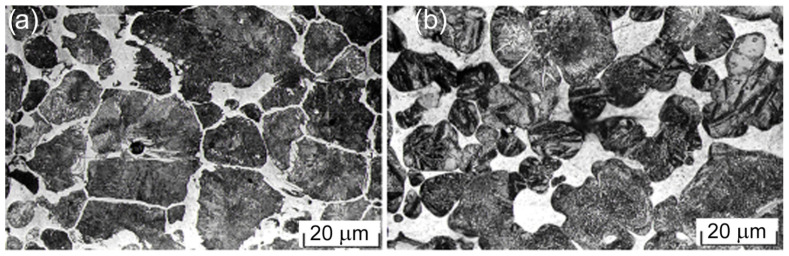
Structure of the material tested: (**a**) initial state, (**b**) quenching with low annealing state.

In the initial state, the microstructure consisted of areas of steel skeleton with pearlite structure and a small amount of cementite, as well as areas of copper phase located on the borders and at the junctures of the skeleton (Figure 1a). The quenching formed a troostite–martensite structure (Figure 1b).

### 2.2. Methods

The turning was carried out with a CNC CKE6136i DMTG lathe using a DCMT11T304-PSS cemented carbide insert of AH8015 grade (Tungaloy, Bielany Wrocławskie, Poland). Inserts were PVD coated with nano-multilayer AlTiN coating. The processing was performed under dry machining because, based on environmental benefits, such conditions are welcomed by manufacturing companies [32]. Ranges of the cutting parameters used when turning are introduced in Table 1. The used values were selected according to the 2^2^ full factorial design plane.

The chip analyzing was performed with a DINO Lite AM7013MZT digital microscope (Dino-Lite Europe, Almere, Netherlands) and JEOL 7600 (JEOL, Warszawa, Poland). Basing on the measurement results, values of the chip spreading ratio were calculated using Equations (1) and (2):

(1) $$K_{b}=\frac{b_{ch}}{\bar{b_{D}}},$$

where $b_{ch}$ is the width of chip and $\bar{b_{D}}$ is the width of undeformed chip.

The average width of the cut $\bar{b_{D}}$ was used to calculate $K_{b}$. During turning with small *a_p_*, the main cutting edge and the nose radius edge were involved in the machining; hence, the relationship was used to calculate the width of undeformed chip:

(2) $$\bar{b_{D}}=\frac{f}{1-\frac{r_{\varepsilon }}{a_{p}}\left[1-\sqrt{1-{\left(\frac{f}{{2r}_{\varepsilon }}\right)}^{2}}\right]}\cdot \sin arc\;tg\cdot \frac{1-\frac{r_{\varepsilon }}{a_{p}}\left[1-\sqrt{1-{\left(\frac{f}{{2r}_{\varepsilon }}\right)}^{2}}\right]}{\left[1-\frac{r_{\varepsilon }}{a_{p}}\left(1-\cos\kappa_{r}\right)\right]\cdot ctg\kappa_{r}+\frac{r_{\varepsilon }}{a_{p}}\left(\sin\kappa_{r}+\frac{f}{{2r}_{\varepsilon }}\right)},$$

where f is the feed, $a_{p}$ is the thickness of the undeformed chip, $r_{\varepsilon }$ is the nose radius, and $\kappa_{r}$ is the tool cutting edge angle.

The surface topography of samples measuring was carried out using a Sensofar S Neon optical profiler (Sensofar, Barcelona, Spain). The parameters analyzed were *Sz* (maximum height of surface), *Sp* (maximum peak height), and *Sv* (maximum valley height).

The tests were repeated 3 times. Statistical analysis was performed using Statistica 13 software.

## 3. Results and Discussion

### 3.1. Chip Shaping

**Figure 2:**
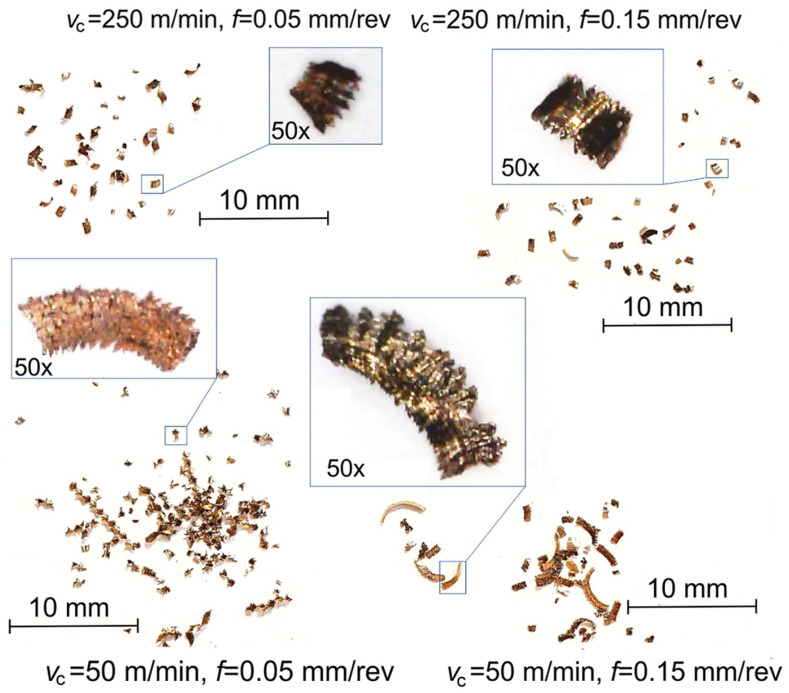
Chip shapes when machining of an initial material.

**Figure 3:**
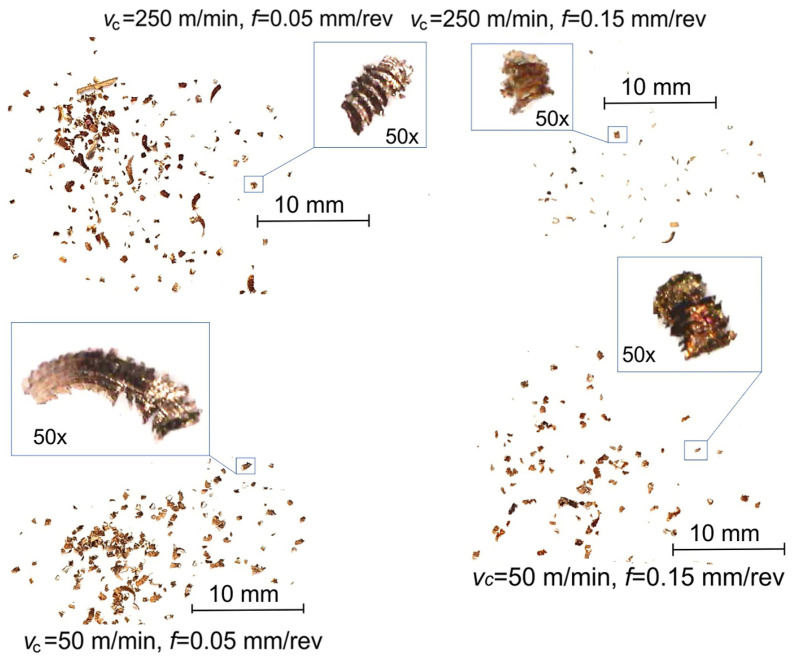
Chip shapes when machining of material after quenching.

The chip shapes for powder steels in the initial state (after infiltration) and after quenching when finish turning depending on the *v_c_* and the *f* are shown in Figure 2 and Figure 3, respectively.

The details of the chip shape are very important to evaluate the changes in the surface texture in the course of machining and the possibility of chip removal, etc. It was found that when finish turning of powder steels, the chips took the shape of small debris and, less frequently, loose curve or element chips (Figure 2). It is possible to identify the fragments of a segmented chip, the length of which was significantly greater with low speeds, while the feed had little effect. Quenching provided the formation of a harder yet more brittle material. Hence, fine chips occurred at a larger range of speeds and feeds, while the segmented sections were shorter (Figure 3). Short, breakable chips were easily evacuated from the cutting zone and their presence led to a better surface quality, as they reduced the risk of surface scratches compared to long and continuous chips [33].

### 3.2. Chip Morphology

**Figure 4:**
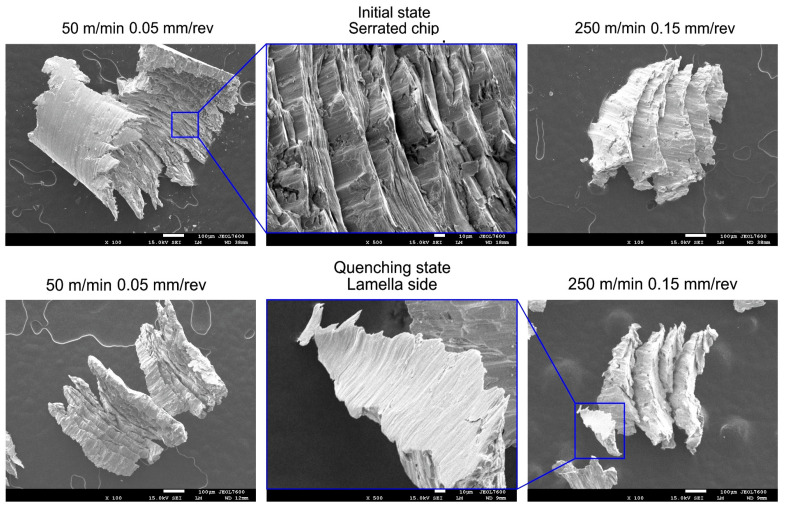
Results of SEM analysis for chip morphology of the materials tested.

Details of the chip morphology based on the scanning microscopy results are shown in Figure 4. Powder steels are materials with an increased brittleness; therefore, changes in the course of the chip formation characteristic of brittle materials can be expected. Depending on the values of *v_c_* and *f*, the chip morphology evolves from continuous to serrated and then to fragmented, as described by Wang et al. [34]. When turning quenched steel, the formation of uneven lamellas is possible, as Günay and Korkmaz [35] revealed. On the other hand, when turning materials in the initial state, serrated chips are formed. From the viewpoints of material removal, chip disposal, and production automation, serrated and fragmented chips are preferable compared to continuous chips.

### 3.3. Changes in Chip Spreading Ratios

**Figure 5:**
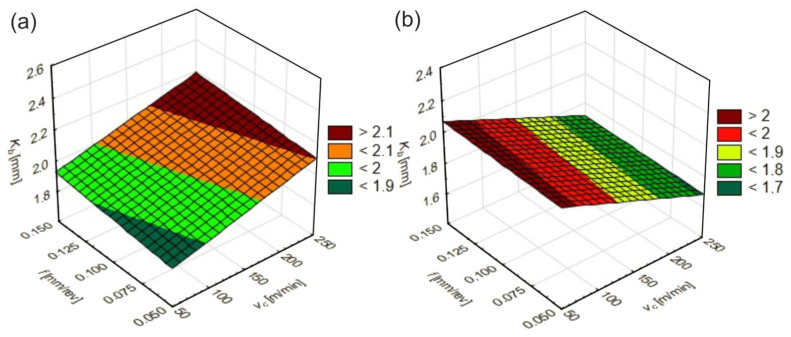
The effect of cutting parameters on *K_b_* values: (**a**) initial state, (**b**) quenching state.

A significant factor characterizing features of a small-size chip formation is chip widening, which can be evaluated based on the chip spreading ratio, *K_b_*. It can be defined as a ratio of the deformed chip width to the width of the undeformed chip. *K_b_* is used to analyze changes in the width of the material during the cutting process and its value decreasing indicates a less intense chip deformation process of the workpiece material. The effect of the turning parameters on *K_b_* is shown in Figure 5.

When turning the materials tested, the cutting speed had greater influence on *K_b_* values. It might be stated that for the initial state material, *K_b_* values increased with an increasing *v*_c_, while the opposite trend was observed for the quenching state. For the initial material, a reduction in *K_b_* values was observed in the range of cutting speeds lower than 50–100 m/min and at feed rates lower than 0.05–0.075 mm/rev, while for the quenched material, this occurred in the range of cutting speeds higher than 225–250 m/min and feed rates lower than 0.05–0.075 mm/rev. The changes observed can be induced by changes in the mechanical and thermal properties of the materials tested. A reduction in the *K_b_* value indicates a reduction in the cutting forces and, therefore, a reduction in the load on the cutting tool and machine tool. Lower cutting forces can favorably affect the quality of the surface machined, minimizing the vibrations and improving the stability of the cutting process [36], and can also lead to a reduction in the dynamic loads on the tool and reduce the tool wear [37].

**Figure 6:**
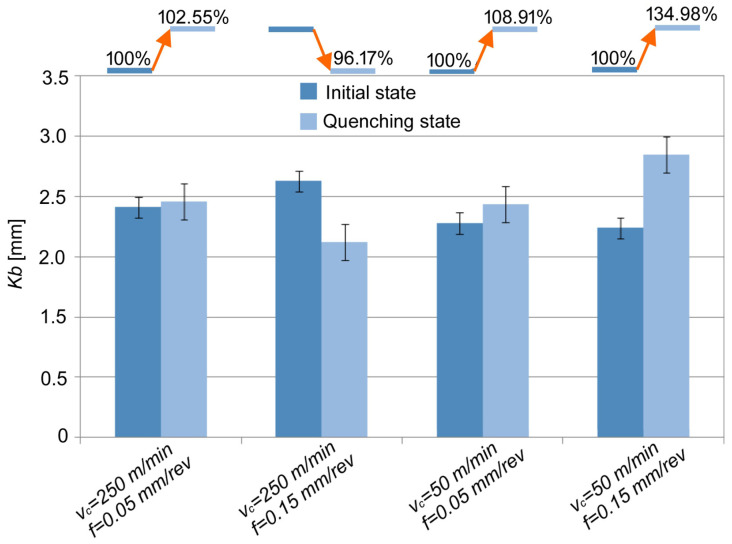
The effect of the materials tested on percentage changes in *K_b_* depending on *v_c_* and *f*.

The effect of the materials tested on the percentage changes in *K_b_* as a function of the *v_c_* and *f* is shown in Figure 6.

A noticeable effect of the cutting parameters and material properties on the *K_b_* values and percentage changes can be observed in Figure 6. Compared to the initial state, a 14% reduction in *K_b_* was observed during turning quenching state at high cutting speeds and feed rates. In other conditions, an increase in *K_b_* in the range of 2 to 35% was observed.

### 3.4. Changes in Surface Roughness Parameters

The *Sz* parameter, which is the sum of the *Sp* and *Sv* parameters, is one of the decisive parameters to control the surface quality [38], particularly in the production of precision mechanical parts, where the surface roughness has a direct impact on their functionality and durability [39].

**Figure 7:**
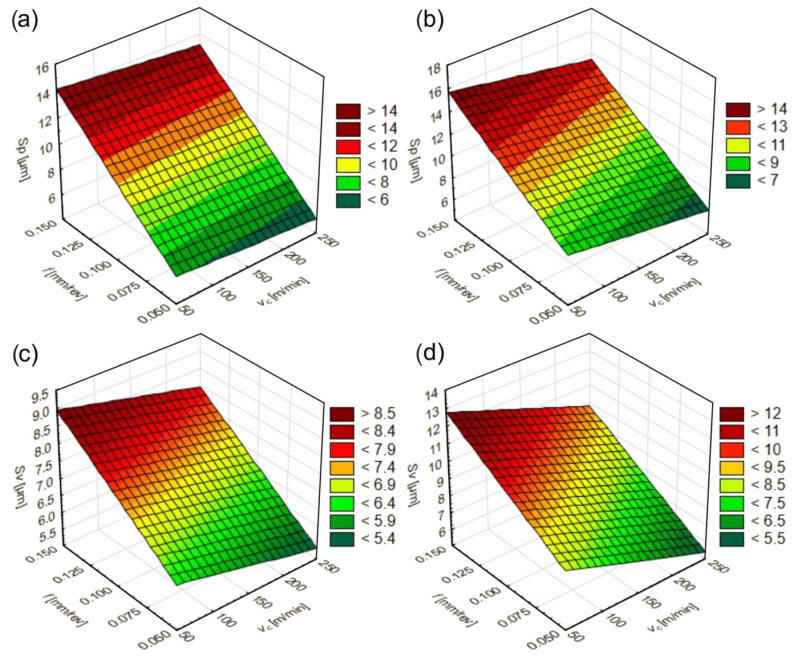
Effect of *v_c_* and *f* on *Sp* and *Sv* parameters for the initial state ((**a**) and (**c**), respectively) and for the quenching state ((**b**) and (**d**), respectively).

The effects of *v_c_* and *f* on *Sp* and *Sv* values when turning the materials tested are shown in Figure 7.

When turning the materials tested, the feed rate had the greatest effect on the *Sp* and *Sv* parameters. As it increased, the *Sp* and *Sv* parameters also increased. For both the initial and the quenching states, a decrease in the height of the highest peak *Sp* and the depth of the lowest valley *Sv* was achieved in the cutting speed range of 200–250 m/min and feed rates of 0.05–0.075 mm/rev. In contrast, increases in these parameters were registered in the ranges of 50–150 m/min and 0.125–0.15 mm/rev.

**Figure 8:**
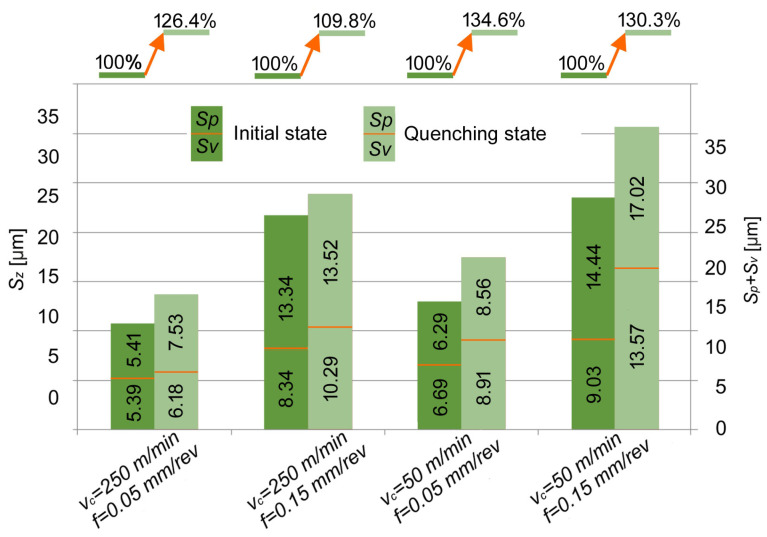
Effect of the materials tested on the percentage changes in *Sz* values as a function of the *v_c_* and *f*.

The effect of the materials tested on the percentage changes in *Sz* values as a function of the *v_c_* and *f* is shown in Figure 8. It is easy to see the distinct effect of the cutting parameters and the workpiece materials. Both the cutting speed and feed rate, as well as an increase the in material hardness, resulted in an increase in the *Sz* parameter of the surface texture. Compared to the initial state, only an increase in the *Sz* parameter in the range of 10 to 35% was observed with the turning quenching state.

### 3.5. Surface Topography Shaping

The surface topography can provide a lot of information to optimize the production processes and ensure the quality of machined parts [40]. By shaping the surface appropriately, high mechanical, tribological, and aesthetic properties of the manufactured products can be achieved. In addition, the surface topography influences the production efficiency of industrial components as well as their service life [41,42].

**Figure 9:**
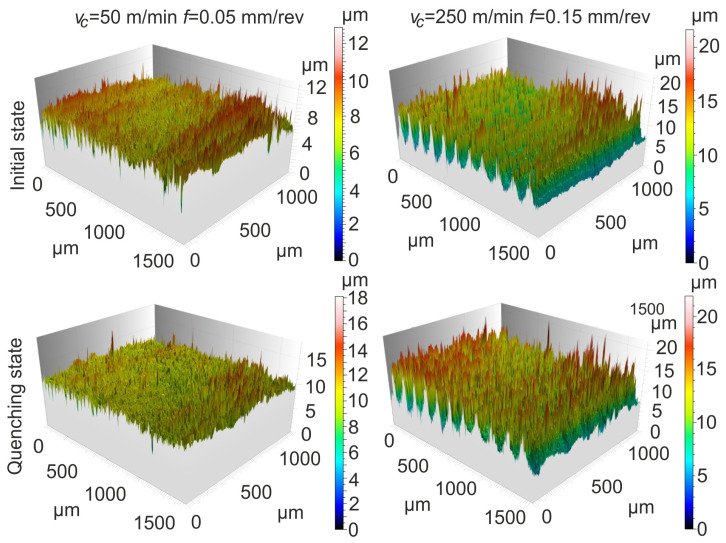
Surface topographies of the tested materials after finishing turning as a function of *v_c_* and *f*.

The surface topographies of the tested materials under finish turning depending on the cutting parameters are shown in Figure 9.

After turning with *v_c_* = 50 m/min and *f* = 0.05 mm/rev, a wave-like shape and single peaks were observed on the surfaces of the materials tested. In the case of the initial material, the heights of surface irregularities reached up to 12 µm, and for the quenching material these reached up to 18 µm. On the surfaces machined with 250 m/min and 0.15 mm/rev, classic feed tracks were observed in the form of valleys and irregular ridges with heights of up to 20 µm. Greater areas of such heights were observed on the surface after quenching. Under higher cutting speeds, the temperature in the cutting zone increases, which can cause local softening of the infiltrated and quenched powder steel, resulting in irregular material removal and the formation of non-uniform ridges. On the other hand, sudden temperature changes can lead to thermal stresses that can deform the surface machined.

Many studies are known to interconnect the features of machined surface topography and wear behavior. Barrell and Priest [43] tested the interaction of a cast iron pin with a hardened spring steel disk with SAE 10W40 lubrication under unidirectional sliding conditions, observing the changes in friction, wear rate, and surface roughness. Such conditions are typical of asperity contact when two rough surfaces are in close proximity. A significant effect of the *Ra* (average roughness of the surface’s measured microscopic peaks and valleys) and *RMS* (root mean square of the surface’s measured microscopic peaks and valleys) was shown. Hadinezhad et al. [44] conducted a pin-on-disk dry test and determined the effects of the slip distance, hardness, and surface roughness on the wear rate and plastic deformations. Son et al. [45] proposed an analytical model of adhesive wear of two unlubricated rough surfaces forming microcontacts under normal load and described the momentary wear volume depending on the material properties, roughness parameters, and loading conditions. Zabala et al. [46] determined the effect of surface roughness on tribological behavior of the lubricated tribosystem steel on steel. An exponential dependence between the *Ra* and *Rt* parameters and wear was found.

Many studies have determined the dependence of the wear of friction surfaces on the surface texture parameters. Based on their analysis, it has been revealed by the authors of this paper that the linear wear of the friction surfaces can be approximated with sufficient accuracy using the following dependence:

*U* = β*S*^α^, (3)

where α and β are parameters of the starting wear curve; and *S* is the friction path, km.

**Figure 10:**
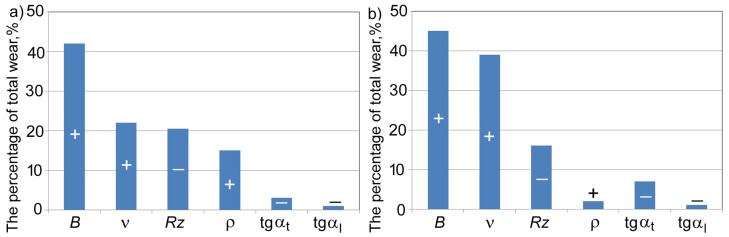
The effect of surface topography parameters on the wear rate (“+” symbol indicates a positive effect on wear, while “–” symbol indicates a negative one).

It was found that the roughness parameters determined 70–80% of variations in the wear resistance indices of α and β of flat surfaces, and that the intensity of this impact was different (Figure 10).

To define the role played by roughness parameters, non-dimensional variables as well as the parameters themselves may be used. So, the equations to calculate the α and β parameters of the starting wear curve for friction of two flat surfaces are as follows

(4) $$\alpha =C_{\alpha }B^{k_{1}}v^{k_{2}}{Rz}^{k_{3}}\rho^{k_{4}};\;\beta =C_{\alpha }B^{n_{1}}v^{n_{2}}{Rz}^{n_{3}}\rho^{n_{4}}$$

where *C*_α_ and *C*_β_ are coefficients; and *k*_1_, *k*_2_, *k*_3_, *n*_4_ > 0 and *k*_4_, *n*_1_, *n*_2_, *n*_3_ < 0 are degree indices.

The non-dimensional variables are

(5) $$B=\frac{RSm}{{Rz}^{v}};\;v=2Rmr\left(50\%\right)\frac{Rp}{Ra}-1\;;\;\rho =\sqrt{\rho_{t}\rho_{1}}$$

where *RSm* is the mean width of the profile steps; *Rmr*(50%) is the material ratio of the profile at 50% level; *Rz* is the maximum profile height; *Rp* is the maximum profile peak height; *Ra* is the arithmetic mean deviation; and ρ_p_ and ρ_w_ are the mean curvature radius for the profile peaks in the transverse (t) and longitudinal (l) directions.

**Figure 11:**
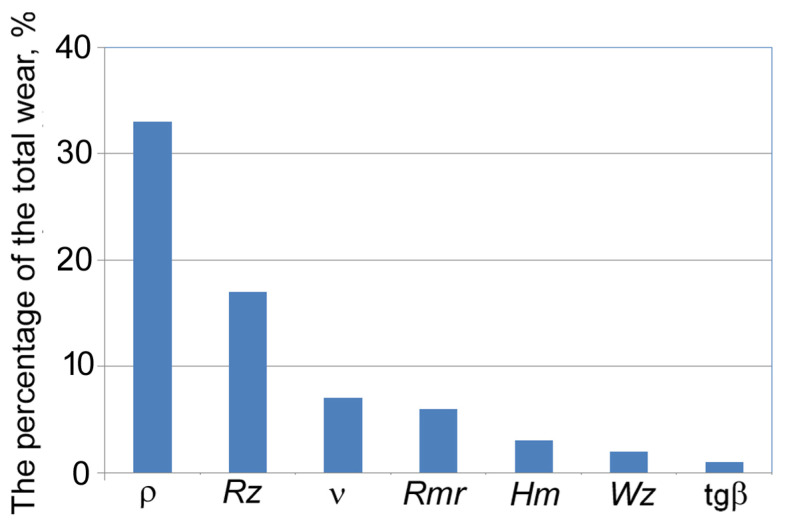
The impact of roughness parameters of cylindrical fiction surfaces on their wear.

Similar results were obtained when studying the wear resistance of cylindrical surfaces (Figure 11). The equations to calculate parameters of the starting wear curve for friction of two cylindrical surfaces are as follows:

(6) $$\alpha =C_{\alpha }b^{m_{1}}{Ra}^{m_{2}}\rho^{m_{3}}H_{µ}^{m_{4}};\;\beta =C_{\beta }b^{p_{1}}\rho^{p_{2}}{Hm}^{p_{3}}$$

where *C*_α_ and *C*_β_ are coefficients; *m*_1_, *m*_2_, *m*_3_, *p*_3_ > 0 and *m*_4_, *p*_1_, *p*_2_ < 0 are degree indices; *H_m_* is the microhardness of friction surfaces; *Ra* is the arithmetic mean deviation; and *Wz* is the maximum waviness height. $\beta =\sqrt{r_{1}r_{2}}$ is the mean geometric radius of curvature of cylindrical surfaces.

According to Equations (3)–(6), the values of roughness parameters are the basis for determining the wear resistance of the surfaces machined. It is possible to transfer such influences for authentic 3D parameters *Sa*, *Sp*, *Sv*, and *Sz* with high probability.

## 4. Conclusions

This research paper discusses the chip and surface texture shaping during finish turning of powder steels infiltrated with tin bronze. The following dependencies have been found:

- When finish turning powder steels, the chips take the shape of small fragments or, less often, loose curves or element chips. When turning quenched steel, the formation of uneven lamellas is possible and, when turning initial steel, serrated chips are formed. Short, breakable chips are easily removed from the cutting zone and their presence leads to a favorable surface quality, as it reduces the risk of scratching. Compared to the initial state, quenching ensures the creation of a harder but more brittle material. As a result, fine chips occur over a wider range of speeds and feed rates and the segmented sections are shorter;
- Changes in the chip spreading ratio *K_b_* indicate a less intensive process of the chip deformation. It was revealed that for the initial material, *K_b_* values increase with increasing *v*_c_, while the opposite trend was observed for the quenched material. For the initial state, a reduction in *K_b_* was observed in the range of a *v_c_* of 50–100 m/min and an *f* of 0.05–0.075 mm/rev, while for quenched material this was in the range of 225–250 m/min and 0.05–0.075 mm/rev. Compared to the initial state, a 14% reduction in *K_b_* was registered for quenching at high cutting speeds and feed rates, and up to a 32% reduction was observed under other conditions;
- When turning the tested materials, the feed rate had the greatest effect on the *Sp* and *Sv* texture parameters. As it increased, the *Sp* and *Sv* parameters also increased. For the initial state and after quenching, a decrease in the *Sp* and *Sv* parameters was achieved in the range of *v_c_* of 200–250 m/min and *f* of 0.05–0.075 mm/rev, and an increase was achieved in the range of 50–150 m/min and 0.125–0.15 mm/rev. Both the cutting speed and feed rate, as well as an increase in the material hardness, resulted in an increase in the *Sz* parameter of the surface texture. Compared to the initial state, an increase in the *Sz* parameter from 10 to 35% was observed for quenching;
- When turning with a *v_c_* of 50 m/min and an *f* of 0.05 mm/rev, wave-like formations with single peaks were observed on the surfaces of the tested materials. The heights of these surface irregularities reached up to 12 µm for initial state and 18 µm after quenching. On the surfaces machined with 250 m/min and 0.15 mm/rev, classic feed marks in the form of valleys and irregular ridges with heights of up to 20 µm were observed. The results of the investigations can be useful when designing and manufacturing industrial parts made of power steels.

## Figures and Tables

**Table 1:** Cutting parameters used.

*a*_p_, mm	*f*, mm/rev		*v*_c_, m/min	
	Value	Code	Value	Code
0.25	0.05	−1	50	−1
	0.15	+1	50	−1
	0.05	−1	250	+1
	0.15	+1	250	+1

## Data Availability

The raw data supporting the conclusions of this article will be made available by the authors on request.
